# Transient bacteremia during coblation-assisted tonsillotomy in children

**DOI:** 10.55730/1300-0144.6367

**Published:** 2026-06-25

**Authors:** Yüce İSLAMOĞLU, Sami BERÇİN, Bengi ARSLAN, Gökhan ATİK, Mehmet Emin AK, Muhammed Fatih ÖZCAN, Selman SEÇKİN, Ezgi ERKILIÇ, Bedia DİNÇ

**Affiliations:** 1 Department of Otolaryngology – Head and Neck Surgery, Ankara City Hospital, Faculty of Medicine, Health Sciences University, Ankara, Turkiye; 2 Department of Otolaryngology – Head and Neck Surgery, Faculty of Medicine, Ankara Yıldırım Beyazıt University, Ankara, Turkiye; 3 Department of Otolaryngology – Head and Neck Surgery, Ankara City Hospital, Ankara, Turkiye; 4 Department of Anesthesiology and Reanimation, Ankara City Hospital, Faculty of Medicine, Health Sciences University, Ankara, Turkiye; 5 Department of Microbiology, Ankara City Hospital, Faculty of Medicine, Health Sciences University, Ankara, Turkiye

**Keywords:** Tonsillotomy, intracapsular partial tonsillectomy, coblation, bacteremia, pediatric otolaryngology

## Abstract

**Background/aim:**

The aim of this study was to evaluate the incidence of transient bacteremia after coblation-assisted intracapsular partial tonsillectomy (tonsillotomy) in children.

**Materials and methods:**

This prospective study included 42 children with grade 3–4 tonsillar hypertrophy who underwent coblation-assisted tonsillotomy for obstructive sleep apnea syndrome. Aerobic and anaerobic blood cultures were obtained preoperatively and immediately after tonsillotomy. Intraoperative throat cultures were also obtained before mucosal manipulation.

**Results:**

All preoperative blood cultures were negative. Postoperative blood cultures were positive in two patients (4.8%; 95% CI, 0.6%–16.2%). *Streptococcus pyogenes* and *Actinomyces* spp. were isolated. The patient with *S. pyogenes* bacteremia also had *S. pyogenes* in the corresponding throat culture. No clinical complications related to transient bacteremia were observed.

**Conclusion:**

This is the first study on evaluation of transient bacteremia after coblation-assisted tonsillotomy. Although the technique is minimally invasive, transient bacteremia occurred in 4.8% of the children. Antibiotic prophylaxis should be considered individually in patients at high risk for bacteremia-related complications, in accordance with current recommendations.

## 1. Introduction

Tonsillectomy is one of the most frequently performed procedures in otorhinolaryngology. Although extracapsular tonsillectomy has traditionally been the standard technique, intracapsular partial tonsillectomy, also known as tonsillotomy, has gained increasing acceptance in pediatric practice with advances in surgical instrumentation [1,2].

Tonsillotomy is particularly useful in children with obstructive tonsillar hypertrophy because it is associated with less postoperative pain and a lower risk of postoperative hemorrhage than conventional extracapsular tonsillectomy [3,4]. Consequently, its use has increased in recent years.

Transient bacteremia may occur after tonsillectomy [5–8]. This finding is clinically relevant because bacteremia-related complications may be important in selected high-risk patients, including those with certain cardiac conditions or prosthetic material [9].

During tonsillectomy, disruption of the oropharyngeal mucosa and the vascular tonsillar bed may allow bacteria from the heavily colonized surgical field to enter the bloodstream [7,8]. In tonsillotomy, mucosal and capsular injury is more limited and coblation technology may further reduce collateral thermal tissue damage [10].

However, whether the increasingly used tonsillotomy procedure is associated with transient bacteremia has not been specifically evaluated previously.

The aim of the present study was to determine whether transient bacteremia occurs in pediatric patients undergoing coblation-assisted tonsillotomy and to assess its incidence. We hypothesized that this technique would be associated with a lower rate of transient bacteremia than reported for conventional tonsillectomy.

## 2. Materials and methods

### 2.1. Study design and ethical approval

This prospective study was approved by the Ankara City Hospital Medical Research and Ethics Committee (approval date: 11 June 2025; approval number: TABED 2/25/1132/2025). Written informed consent was obtained from the parents or legal guardians of all participants.

### 2.2. Participant selection and statistical analysis

The study was designed to evaluate the presence and frequency of transient bacteremia following coblation-assisted tonsillotomy in pediatric patients. Given the absence of prior published data regarding bacteremia associated with this specific surgical technique, a formal power calculation was not feasible. Consequently, a prospective cohort of 42 consecutive patients was enrolled to provide preliminary safety and feasibility data, serving to inform future larger-scale investigations.

Descriptive statistics were used to summarize the study data. Continuous variables were expressed as mean ± standard deviation (SD) or median with interquartile range (IQR), depending on the normality of data distribution, while categorical variables were presented as frequencies and exact proportions. Since no bacterial growth was observed in preoperative blood cultures, formal comparative hypothesis testing between the preoperative and postoperative periods was not performed. Instead, the statistical analysis focused on the postoperative incidence of transient bacteremia. To evaluate the precision of this observed rate, the 95% confidence interval (CI) for the postoperative bacteremia proportion was calculated using the exact Clopper–Pearson binomial method based on the final sample size (n = 42). With this sample size and an assumed true bacteremia rate of approximately 5%, the 95% exact Clopper–Pearson confidence interval was determined to range from 0.6% to 16.2%.

All statistical analyses were performed using SPSS, version 30.0 (IBM Corp. Armonk, NY, USA).

### 2.3. Clinical and demographic data collection

Forty-two children scheduled for coblation-assisted intracapsular partial tonsillectomy were enrolled. The study population included 12 girls and 30 boys, with a mean age of 7.5 years (range 3–12 years). All patients had grade 3–4 tonsillar hypertrophy according to the Brodsky scale. The surgical indication was obstructive sleep apnea syndrome. Patients with chronic tonsillitis, chronic adenoiditis, recurrent sinusitis, or any infection within the preceding month and high-risk patients, including those with certain cardiac conditions or prosthetic material, were excluded.

Before surgery, all patients underwent a clinical evaluation to confirm the absence of upper respiratory tract infection or systemic infection.

All patients were hospitalized for 24 h postoperatively for close monitoring. Vital signs including body temperature, heart rate, and blood pressure, along with oral intake, were regularly assessed. All patients in the study group achieved adequate oral intake and were successfully discharged on the first postoperative day. Follow-up visits were scheduled for the first postoperative week, and patients were subsequently monitored during consecutive weeks for a total duration of approximately 2 months.

### 2.4. Collection of blood and throat cultures

During routine preoperative blood sampling for anesthesia, aerobic and anaerobic blood cultures were obtained for the study. After induction of general anesthesia and before surgical manipulation of the tonsils, a sterile throat swab culture was collected with care to avoid contamination.

Tonsillotomy was performed using the coblation system (Smith & Nephew, London, UK). The procedure was executed utilizing a dedicated wand that automatically calibrates to the manufacturer’s optimal preset ablation and coagulation parameters, thereby minimizing human error and ensuring standardized energy delivery. During the procedure, the tonsillar tissue was systematically reduced down to the level of the anterior tonsillar pillar. This specific controlled technology allows for precise tissue dissolution at lower temperatures, resulting in significantly less collateral thermal damage to the surrounding healthy tissues. Immediately after completion of the tonsillotomy and before any additional procedures, the anesthesia team obtained postoperative aerobic and anaerobic blood cultures under sterile conditions.

In patients requiring adenoidectomy or ventilation tube insertion, these procedures were performed only after postoperative blood cultures had been collected to avoid confounding the bacteremia results. All patients subsequently underwent adenoidectomy for adenoidal obstruction and ventilation tubes were inserted in five patients.

### 2.5. Analysis of blood and throat cultures

Both preoperatively and intraoperatively, blood samples were collected in paired BACT/ALERT culture bottles including one aerobic and one anaerobic bottle per patient, with a minimum volume of 5 mL of blood inoculated into each bottle, in accordance with standard institutional blood culture protocols. All samples were subsequently incubated in the BACT/ALERT 3D automated microbial detection system (bioMérieux, Marcy-l’Étoile, France).

Bottles indicating positive growth during the 5-day incubation period underwent Gram staining. Samples were subcultured on 5% sheep blood agar and incubated at 37 °C in a 5% CO_2_ atmosphere for 24 h. Bacterial identification was performed using VITEK MS with MALDI-TOF technology. Antimicrobial susceptibility testing of clinically significant isolates was performed using the VITEK 2 automated system according to the manufacturer’s recommendations.

## 3. Results

No growth was detected in any preoperative blood culture.

*Streptococcus pyogenes* was isolated from intraoperative throat cultures in five patients.

Among the postoperative blood cultures obtained immediately after tonsillotomy, transient bacteremia was detected in two patients (4.8%; 95% CI, 0.6%–16.2%). *S. pyogene*s was isolated in one patient and *Actinomyces* spp. were isolated in the other. The patient with *S. pyogenes* bacteremia was also positive for *S. pyogenes* in the corresponding throat culture.

No postoperative clinical complications attributable to surgery or transient bacteremia were observed in any patient during the 2-month follow-up period.

## 4. Discussion

In the present prospective study, coblation-assisted tonsillotomy was associated with a postoperative positive blood culture rate of 4.8%. To the best of our knowledge, this is the first study to evaluate transient bacteremia specifically after tonsillotomy.

Previous studies about bacteremia following conventional tonsillectomy have shown substantially higher rates, ranging from 19% to 60%, depending on the specific surgical technique, patient population, and blood culture protocol [11–14]. A summary of these historical data is provided in the Table.

Adenoidectomy and ventilation tube insertion have also been reported to cause transient bacteremia [15,16]. To minimize confounding, these procedures were deliberately performed only after the tonsillotomy had been completed and after postoperative blood cultures had been obtained.

The palatine tonsils are heavily colonized tissues. Therefore, any surgical procedure involving the tonsils may theoretically lead to bacteremia through injury to the venous channels within the tonsillar tissue and oropharyngeal mucosa [8,10,11].

The lower bacteremia rate observed in the present study may have been related to the intracapsular nature of tonsillotomy and the use of coblation, which may limit mucosal, capsular, and vascular injury compared with extracapsular techniques [2,10]. However, the occurrence of bacteremia despite these potential advantages indicates that tonsillotomy does not completely eliminate the risk of bacterial translocation into the bloodstream.

Another noteworthy finding was the isolation of *Actinomyces* spp. in a single postoperative blood culture. *Actinomyces* species are well-established commensals of the tonsillar crypts, as frequently documented in histopathological tonsillectomy specimens [17]. Distinguishing true bacteremia from specimen contamination requires a careful evaluation of microbiological criteria, including the number of positive bottles and the time to positivity (TTP). In the present study, blood sampling was performed using strict sterile techniques with both aerobic and anaerobic bottles. The organism was isolated from both the aerobic and anaerobic bottles, with a TTP of 48 h. While the negative preoperative culture and rigorous sampling technique indicate transient bacteremia as a plausible explanation, a single positive culture bottle inherently raises the probability of contamination. Ultimately, a definitive distinction between true bacteremia and contamination cannot be established from a single positive culture. This single isolation remains a study limitation and should be interpreted with caution in an asymptomatic pediatric patient.

Our study has several limitations. First, the sample size was relatively small and the study was conducted at a single center. Second, there was no control group undergoing conventional tonsillectomy. Third, all patients had concomitant adenoidal hypertrophy; however, patients with chronic adenoiditis or recurrent sinonasal infections were excluded and adenoidectomy was performed only after blood culture sampling. These measures minimized the potential effect of adenoidal disease on the bacteremia results.

Larger multicenter prospective studies with control groups and more diverse patient populations are needed to confirm these preliminary findings and to clarify whether antibiotic prophylaxis is warranted in selected high-risk patients undergoing tonsillotomy.

## 5. Conclusion

The present study provides the first data on transient bacteremia after coblation-assisted tonsillotomy in children.

Despite the minimally invasive nature of coblation-assisted tonsillotomy, transient bacteremia occurred in 4.8% of the patients in our cohort. While this risk appears lower than historical rates reported for conventional tonsillectomy, a finding that warrants confirmation in a future controlled trial, bacteremia is notably not entirely absent. Consequently, tailored antibiotic prophylaxis should be considered on an individual basis for patients at high risk for bacteremia-related complications, in accordance with current clinical guidelines. This high-risk subpopulation includes individuals with prosthetic cardiac valves, a history of infective endocarditis, specific congenital heart diseases, postcardiac transplant valvulopathy, or orthopedic joint prostheses [9]. For these cases, a multidisciplinary approach should be adopted, and the prophylaxis strategy should be planned in collaboration with a cardiologist.

## Figures and Tables

**Table:** Comparison of the present study with previous studies showing bacteremia after tonsillar surgery.

Reference	Method	Number of patients	Bacteremia rate (%)
[5]	Tonsillectomy, dissection-snare	40	25
[7]	Tonsillectomy	32	34.4
[11]	Guillotine tonsillectomy	30	60
[11]	Dissection tonsillectomy	21	19
[12]	Guillotine tonsillectomy	32	21.8
[12]	Dissection tonsillectomy	32	28.1
[13]	Dissection tonsillectomy	32	37.5
Present study	Coblation-assisted tonsillotomy	42	4.8
